# A Meta-Analysis: Whether Repetitive Transcranial Magnetic Stimulation Improves Dysfunction Caused by Stroke with Lower Limb Spasticity

**DOI:** 10.1155/2021/7219293

**Published:** 2021-11-28

**Authors:** Yu Liu, Hong Li, Jun Zhang, Qing-qing Zhao, Hao-nan Mei, Jiang Ma

**Affiliations:** ^1^Department of Rehabilitation Medicine, Shijiazhuang People's Hospital, Shijiazhuang 050030, Hebei, China; ^2^Rehabilitation District of Taihe Hospital, Shiyan 442000, Hubei, China; ^3^School of Nursing and Rehabilitation, North China University of Science and Technology, Tangshan 063210, Hebei, China

## Abstract

**Objective:**

To evaluate the efficacy of repetitive transcranial magnetic stimulation (rTMS) in improving lower limb spasticity after stroke.

**Methods:**

The PubMed, Web of Science, Cochrane Library, EMBASE, China National Knowledge Infrastructure (CNKI), China Biology Medicine (CBM) disc, China Science and Technology Journal Database (VIP), and Wanfang databases were searched online from their inception to May 2021 for randomized controlled trials (RCTs) involving repetitive transcranial magnetic stimulation for lower extremity spasticity after stroke. Valid data were extracted from the included literature, and the quality evaluation was conducted with the Cochrane Handbook for Systematic Reviews of Interventions along with the Physiotherapy Evidence Database scale (PE-Dro scale). The data that met the quality requirements were systematically analysed using Review Manager 5.4 software.

**Results:**

A total of 554 patients from seven articles (nine studies) were quantitatively analysed. Outcomes included the Modified Ashworth Scale (MAS), Fugl–Meyer Assessment of Lower Extremity (FMA-LE), Modified Barthel Index (MBI), and Timed Up and Go (TUG), measured as the effect of rTMS compared with controls conditions after treatment. The systematic review showed that rTMS reduced MAS and increased MBI scores, respectively (SMD = −0.24, 95% CI [−0.45, −0.03], *P* = 0.02; MD = 6.14, 95% CI [−3.93,8.35], *P* ＜ 0.00001), compared with control conditions. Low-frequency rTMS (LF-rTMS) significantly improved FMA-LE scores (SMD = 0.32, 95% CI [0.13, 0.51], *P* = 0.001). However, there was no significant difference in FMA-LE scores when using high-frequency rTMS (HF-rTMS) (*P* *>* 0.1) and in TUG times (*P* > 0.1) between the treatment and control groups.

**Conclusions:**

rTMS was effective in improving spasticity and activities of daily living. LF-rTMS has positive clinical effects on enhancing motor function in patients who experience lower extremity spasticity after stroke. To better validate the above conclusions, more multicentre, high-quality, and double-blind randomized controlled trials are needed.

## 1. Introduction

Stroke is a common disease worldwide and causes serious disabilities for patients. More than two-thirds of stroke survivors develop poststroke sequelae that involve impairment of motor function, balance, gait, and activities of daily living [1, 2]. Poststroke spasticity (PSS) is a common motor dysfunction after stroke that clinically manifests as increased muscle tone, positive pathological signs, and tendon hyperreflexia [3], with a prevalence from 4% to 42.6% [2]. Current management of poststroke spasticity has shown that although drug therapy (such as botulinum toxin injection, oral baclofen, dantrolene, sodium, and tizanidine) is effective for improving spasticity and widely used in clinical practice, it had side effects and produced unsatisfied clinical effects such as muscle weakness [4]. Nondrug therapy, such as neuromuscular electrical stimulation and physical therapy, temporarily relieved poststroke spasticity and motor dysfunction. However, some of these interventions demand active participants to become involved, and the duration of the efficacy was relatively short [5, 6]. rTMS has been gradually applied in the clinical treatment of poststroke dysfunction due to its noninvasiveness and safety on the basis of conventional treatment of stroke sequelae. But most of the research focused on motor dysfunction, cognitive disorder, aphasia, and so on. There are few studies on the application of rTMS in poststroke spasticity, and the mechanism is unclear. What is more, rTMS has a significant impact on public acceptance due to the relatively high clinical costs and being excluded in the health insurance in some cities [7–9]. A recent meta-analysis explored the use of rTMS in stroke patients. Two meta-analyses published by McIntyre et al. [10] and Peng et al. [11] analysed the effect of rTMS in the rehabilitation of spasticity after stroke. However, they did not include RCTs for the treatment of lower limb spasticity after stroke, and some new RCTs have been published since then. Moreover, the efficacy of repetitive transcranial magnetic stimulation in improving lower limb spasticity after stroke remains unknown. As a result, the purpose of this study was to perform a systematic review of RCTs that explored the efficacy of rTMS in treating patients with lower limb spasticity after stroke.

## 2. Methods

### 2.1. Literature Search Strategy

We performed a search in the PubMed, Web of Science, Cochrane Library, EMBASE, CNKI, CBM, VIP, and Wanfang databases published up to May 2021. The search terms were “stroke” OR “hemiplegia” OR “cerebrovascular accident” OR “ischemic stroke” OR “hemorrhagic stroke” OR “CVA” OR “apoplexy” AND “repetitive transcranial magnetic stimulation” OR “rTMS” OR “transcranial magnetic stimulation” OR “TMS” AND “spasticity” AND “lower limb” OR “lower extremity”.

### 2.2. Inclusion and Exclusion Criteria

The relevant articles were selected based on the following eligibility criteria: (1) the involved patients were clinically diagnosed with lower limb spasticity after stroke by relevant examinations; (2) the experimental group used rTMS and traditional physical therapy, while the control group underwent traditional physical therapy plus sham rTMS (or only with traditional physical therapy); (3) the outcome measures included the MAS, FMA-LE, MBI, and TUG; and (4) the included articles were RCTs.

Articles meeting with the following criteria were excluded: (1) total sample size of fewer than 10 participants in each study; (2) study with incomplete data; (3) meta-analysis, case report, literature review, guidelines, dissertation, and others; and (4) non-RCTs.

### 2.3. Literature Screening and Data Extraction

Two researchers independently searched and screened the literature based on the above search strategy and removed the studies that did not meet the predefined criteria by reading the abstracts and full texts. Any inconsistencies between the two authors were resolved by discussion or in consultation with the third author. The following data were extracted: study characteristics (authors, year of publication, study design, sample sizes, age, and course of disease) and intervention details (intervention measures, treatment time, stimulated sites, treatment parameters, and outcome measures).

### 2.4. Literature Quality Evaluation

The quality of the included articles was evaluated by two authors using the Cochrane Handbook for Systematic Reviews of Interventions [12] and PE-Dro [13]. In cases of disagreement, a third person made the final decision. Three levels (level A, level B, and level C) were used to rank the quality of each study when using the former method [12]. Regarding the PE-Dro, 0–3 points indicated low quality, 4–7 points indicated medium quality, and 8–11 indicated high-quality [14].

### 2.5. Data Synthesis and Statistical Methods

The outcomes in both the treatment and control groups after the intervention period were extracted. The results were shown by the histogram in one study [15], and we estimated the results based on the *X* and *Y* axes and the corresponding parameters. A six-point scale (0, 1, 1^+^, 2, 3, and 4) was denoted as the MAS scale [16]. To quantify the score for analysis, we calculated 1^+^ as 1.5. If the results were not presented as the means and standard deviations, we calculated the original data using SPSS 25.0 [17] or the method of Wan et al. [18]. The data from the first phase for both groups were extracted in randomized controlled crossover studies [15, 19, 20]. If the variable between two groups in an article was only rTMS, we divided the one article into two studies [17, 21]. Quantitative analysis was performed using Review Manager version 5.4 by two authors. Concerning the continuous variables (excluding *H*_max_/*M*_max_), the mean difference (MD) or the standardized mean difference (SMD) with 95% CI were calculated for the outcome. The heterogeneity among the included studies was assessed by the *χ*^2^ test and Higgins *I*^*2*^ values. If there was clear heterogeneity (*I*^2^ > 50% or *P* < 0.1), a random effects model was used. Otherwise, a fixed effects model was applied.

## 3. Results

### 3.1. Characteristics of the Studies

**Figure 1:**
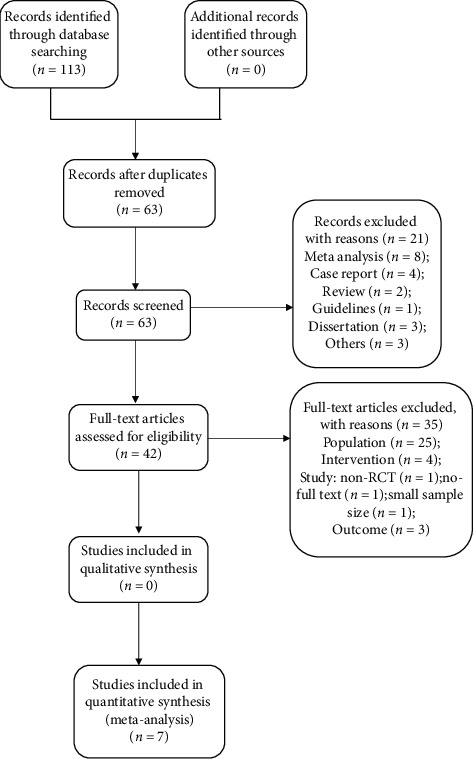
Flow diagram of study selection.

**Figure 2:**
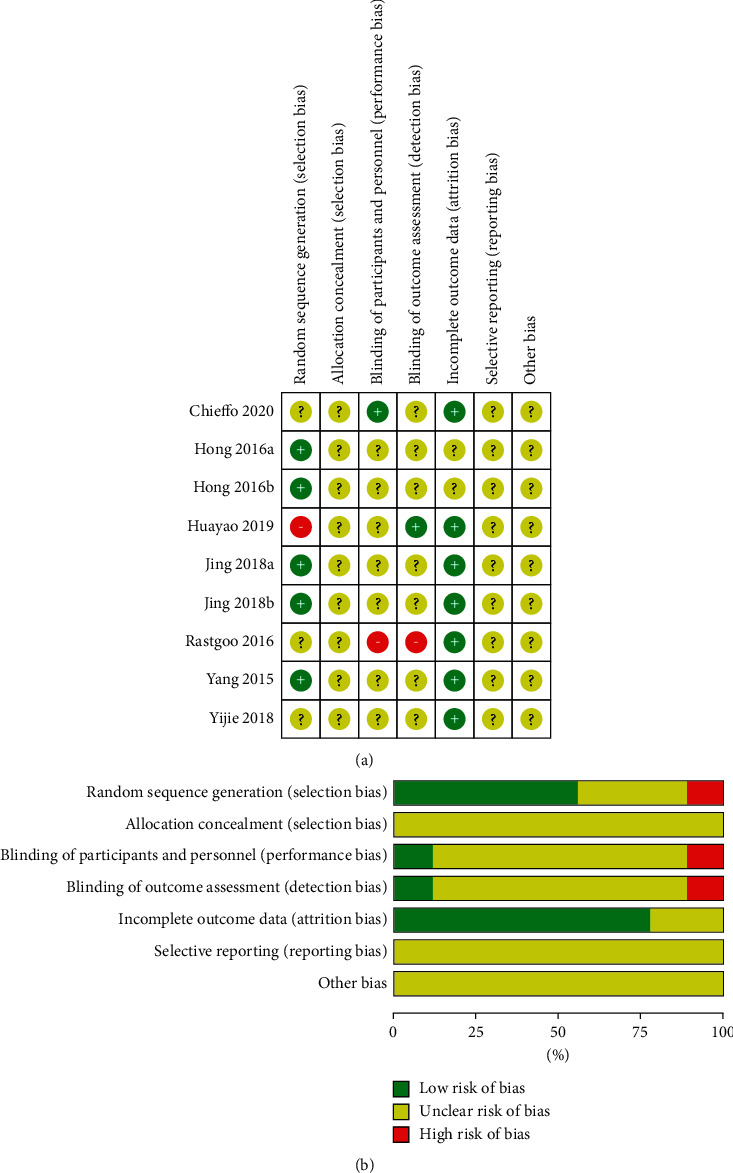
(a) Risk of bias graph. (b) Risk of bias summary.

A total of 113 entries were retrieved from Chinese and English databases, including 33 in Chinese and 80 in English (Figure 1); 50 duplicates were removed through EndNote X9; and 63 articles were screened. Then, 21 were excluded because the article type was not a clinical trial. A total of 42 full-text studies were obtained for eligibility. Then, 35 studies were rejected: 25 due to the population, 4 owing to the intervention, 3 because of the study design, and 3 because of the assessed outcome. Finally, seven articles with a total of 554 patients [15, 17, 19–23] were included. Two articles had two separate data sets [17, 21], and the others had one data set each.  Table 1 shows the characteristics of the included articles. All the included studies were randomized controlled trials with quality level B, and three of them were crossover trials [15, 19, 20]. The risk of bias of the included RCT is shown in Figure 2. The total score on the PE-Dro was 51, with an average of 7.29. Two articles were of high quality [17, 19], and five were of medium quality (Table 2).

### 3.2. Effects of rTMS on Spasticity of the Lower Limbs in Stroke Patients

#### 3.2.1. MAS

**Figure 3:**
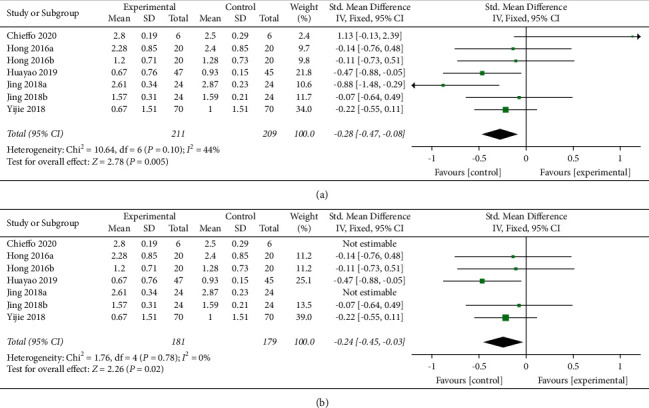
(a) Forest plot for MAS scores. (b) Forest plot for MAS scores when excluding two studies with high sensitivity.

Five articles (seven studies) [17, 19, 21, 23] with 420 patients were included. The forest plot (Figure 3(a)) shows that statistical heterogeneity was not observed (*I*^*2*^ = 44%, *P* = 0.10). We used the fixed effects model because heterogeneity was not observed after two studies were [19, 21] excluded through sensitivity analysis (*I*^*2*^ = 0%, *P* = 0.78). The meta-analysis showed that rTMS had a significant beneficial effect on MAS scores in patients with lower limb spasticity after stroke (SMD = −0.24, 95% CI [−0.45, 0.03], *P* = 0.02) (Figure 3(b)).

### 3.3. Effects of rTMS on Spasticity of the Lower Limbs in Stroke Patients

#### 3.3.1. FMA

**Figure 4:**
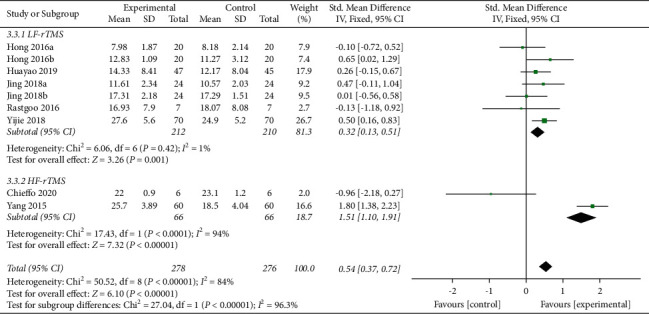
Forest plot of FMA.

A total of seven articles (nine studies) with 554 patients [15, 17, 19–23] presented effects on the FMA. Subgroup analysis based on low- and high-frequency indicated seven studies with LF-rTMS and two with HF-rTMS. The difference between groups among those using low-frequency rTMS showed a statistically significant effect on FMA scores (SMD = 0.32, 95% CI [0.13, 0.51], *P* = 0.001) with no statistical heterogeneity (*I*^*2*^ = 1%, *P* = 0.42). There was no statistical significance between the two groups in the studies using high-frequency rTMS (*P* = 0.72) (Figure 4).

### 3.4. Effects of rTMS on Spasticity of the Lower Limbs in Stroke Patients

#### 3.4.1. MBI

**Figure 5:**
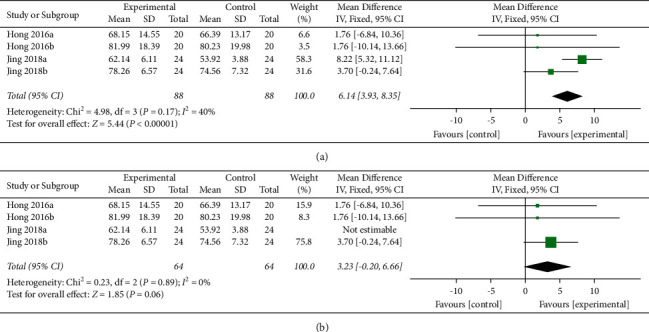
(a) Forest plot for MBI scores. (b) Forest plot for MBI scores after removing one study.

Two articles (four studies) [17, 21] with 176 patients assessed this outcome. The random effects model was used with *I*^*2*^ = 40% and *P* = 0.17 (Figure 5(a)). Jing was the source of the heterogeneity after sensitivity analysis (Figure 5(b)). We found that there was a significant difference between the two groups (MD = 6.14, 95% CI [3.93, 8.35], *P* ＜ 0.00001).

### 3.5. Effects of rTMS on Spasticity of the Lower Limbs in Stroke Patients

#### 3.5.1. TUG Scores

**Figure 6:**
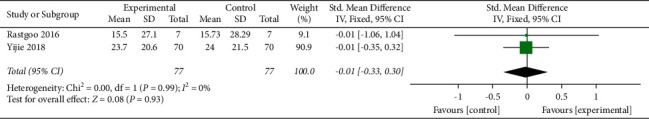
Forest plot for TUG times.

Two studies [15, 20] showed that rTMS did not have a significant effect on MBI scores in the patients with lower limb spasticity (Figure 6).

### 3.6. Others

The electrophysiological index *H*_max_/*M*_max_ was described in two studies [15, 20] and showed that the index decreased in the treatment group without a significant difference compared with the control group.

The included articles reported adverse effects except for Hong [17]. Three studies [15, 20, 21] showed that patients had good tolerance to LF-rTMS in their studies. Chieffo et al. [19] reported transitory dizziness and muscle twitches in the shoulders of three patients, and they subsequently completed the remaining treatment after the intensity was decreased to 80% RMT. In another study, the patients had muscle pain and fatigue symptoms that were relieved after two to three days [21]. Huayao et al. [23] reported adverse effects without a significant difference between the two groups. In the treatment group, transitory headache was found in two patients, which diminished after suspension of the treatment [22].

## 4. Discussion

Velocity-dependent increases in muscle tone, hyperexcitable stretch reflexes, and hyperreflexia tendon jerks are often described as features of spasticity that appear in patients with stroke [24]. The reason for muscle spasticity in stroke patients is the hyperreflexia of the stretch reflex from the spine. The excitability and inhibitory imbalance of spinal descending fibres was considered the main cause of hyperreflexia of the stretch reflex. The disorder presents with high excitability of the reticular spine [25]. In addition, there has also been evidence that the vestibular spinal cord was less inhibitory [26], which reduces the inhibition of the spinal cord. Stroke patients with lower limb spasticity generally manifest hip adduction, knee extension, and ankle plantar flexion impacting the recovery of motor function and gait [27].

At present, the conventional treatment for lower limb spasticity after stroke includes drugs, motor therapy, and neuromuscular electrical stimulation [6]. However, the effect of conventional rehabilitation is limited. Thus some ways of complementary and alternative medicine are needed. Previous studies have shown that rTMS can be used to treat patients with lower limb spasticity after stroke with different results and unclear mechanisms. Naghdi et al. [28] reported that improvements in ankle plantar flexor and knee extensor spasticity were significant, but *H*_max_/*M*_max_ showed no statistical improvement after five consecutive LF-rTMS sessions. Terreaux et al. [29] showed that 1 Hz rTMS reduced the excitability of the ankle plantar flexor reflex without modifying clinical signs of spasticity, but there was no change during 10-Hz rTMS. Because these studies were a nonrandomized controlled trial [28] or had a small sample size [29], our research group performed a systematic analysis including the most recent randomized controlled trials with more participants to further increase the quality of the included studies.

The results indicated that rTMS was effective in improving lower limb spasticity and activities of daily living; LF-rTMS had a positive influence on enhancing motor function in patients who experienced lower extremity spasticity after stroke, whereas HF-rTMS did not have a significant effect on motor function. Furthermore, rTMS did not have an additional significant effect on TUG times or *H*_max_/*M*_max_. McIntyre et al. [10] performed a systematic review regarding the use of rTMS in patients with spasticity after stroke and discovered that rTMS improved spasticity in the elbow, wrist, and finger flexors in uncontrolled pre-post studies, whereas there was no significant influence on spasticity in the wrist in two RCTs. Another meta-analysis by Xu et al. [11] was published in 2020 and reported no benefits on the use of rTMS for upper limb spasticity after stroke, which was different from our research.

Sensitivity analyses of the included studies found that the cause of high bias of risk in Jing and Yan [21] may have been the short duration of treatment and the poor baseline condition of the patients. Rastgoo [15] and Yijie [20] reported that the reason why gait function was improved in both the treatment and control groups after the treatment was that patients were similar regarding TUG measures (TUG refers to the time it takes for the subject to stand up after hearing the instruction, walk straight for three meters, and return to the sitting position with the quickest speed [30]). Few of them had barriers to walking. Electrophysiological changes (*H*_max_/*M*_max_) were [31] not accompanied by clinical improvement in spasticity in Rastgoo et al. [15], which was similar to the result of Dos Santos et al. [32]. *H*_max_/*M*_max,_ which has been related to an increase in muscle tone in spasticity to a certain extent, is the ratio of the maximum amplitude of *H* reflexion and *M* wave recorded by surface electromyography. *H*_max_/*M*_max_ does not completely reflect the excitability of neurons with measurement of error, and *H*_SLP_/*M*_SLP_ was proposed as a better outcome [33]. *H*_SLP_/*M*_SLP_ (the ratio of the slope of *H* and *M*-waves) was more sensitive to represent the excitability of motor neuron of the anterior horn of the spinal cord [34]. In addition, the reasons for unsynchronized changes were that the treatment times of rTMS were short with only five sessions and the relationship between *H*_max_/*M*_max_ and MAS is not exact, which may not change with the change of MAS [35]. There were no serious adverse effects reported during the process of treatment. Five studies [15, 17, 20–22] followed up patients after therapy, and Jing [21] reported that FMA, MAS, and MBI scores were significantly different between the treatment and control groups after the 12-month follow-up. rTMS is based on the principle of electromagnetic induction and produces changes at the stimulated site and transsynaptically in distant cortical regions. Sustained physiological effects were a feature of rTMS, namely, long-term potential and long-term depression [36], while neuromuscular electrical stimulation had a short duration of the therapeutic effect without the above features [37], as previously mentioned.

The mechanism by which rTMS improves spasticity is not clear. There are two commonly used models of rTMS: (1) a high-frequency (>1 Hz) facilitatory mode, which is applied to the affected brain region to increase cortical excitability and thus reduce spasticity and improve upper limb motor function, and (2) a low-frequency (≤1 Hz) inhibitory mode, which decreases excitability in the unaffected hemisphere and therefore reduces inhibition from the contralateral hemisphere to the ipsilateral hemisphere [31, 38, 39]. We concluded from the subgroup analysis that the LF-rTMS group showed a significant influence on lower limb motor function, while there were no benefits of HF-rTMS. The better therapy between high- or low-frequency stimulation has been controversial. Based on the guidelines written by Lefaucheur et al. [40], LF-rTMS over the contralesional hemisphere promoted poststroke recovery of motor function in chronic stroke patients. Owing to the limited studies we included, the curative effect of HF-rTMS remains to be discussed. Guo et al. [41] reported that the reorganization of the motor network was found with both HF-rTMS and LF-rTMS, and both improved motor recovery; HF-rTMS had more positive effects on the functional connectivity reorganization of the ipsilesional motor network. In addition to stimulation frequency, the stimulation parameters of rTMS also include stimulation duration and intensity. Further studies about parameters selection that make rTMS optimally effective need to be conducted.

The stimulated site included in this study was the leg motor cortex, which is associated with a deep position that is located on the inner side of the anterior central gyrus under a thick skull [42]. To ensure that rTMS has a good effect on the rehabilitation of patients with lower limb spasticity after stroke, the requirement for stimulation depth was quite high. In addition to the intensity of the rTMS used in the adopted studies (i.e., 80%–90% RMT), the rTMS coil itself also played a pivotal role in the effects of lower limb spasticity after stroke. The types of coils were different across studies included in our analysis: Rastgoo et al. [15] and Yijie [20] used a figure-8-shaped coil with higher focal stimulation and less white matter penetration. Chieffo et al. [19] found that the reason that the use of an H coil with deeper stimulation did not improve spasticity and walking function was that rTMS combined with cycling did not influence the functional networks involved in the coordination of gait and skilled walking. This was also considered the reason for the heterogeneity observed in this meta-analysis. Although the circular coil had deep penetration, the short duration of treatment with unconcentrated stimulation was the reason for the lack of improvements in lower limb MAS scores [17].

The limitations of this study are as follows: first, the risk of bias was slightly high. The three crossover trials included in this paper only contributed data from the first stage to this analysis; thus, the testing power was reduced. In addition, the affected region of the brain is a factor that influences spasticity after stroke, and subgroup analyses of the main muscle groups in the context of lower extremity spasticity were not conducted. The MAS, FMA, and MBI are semiquantitative indicators, and the evaluators may have introduced subjectivity when assessing them. More large-scale and high-quality randomized controlled trials with targeted and precise quantitative indicators as outcomes are expected in the future.

In summary, rTMS was effective in improving spasticity and activities of daily living in patients with lower limb spasticity after stroke. LF-rTMS had a positive effect on enhancing motor function.

## Figures and Tables

**Table 1:** Characteristics of included articles.

Author/year	Sample size	Mean age (y)	Course of disease (m)	Intervention	Treatment duration	Stimulated site	Treatment parameters	RCT type	Outcome
	Treatment/control								
Chieffo et al., 2020 [19]	6/6	58.67 ± 10.33/61.17 ± 8.70	41.50 ± 26.77/41.00 ± 26.54	*r* + *c*/sham + *c*	15 min/day, 11 sessions	Bilateral leg motor cortex	20 Hz, 80%–90% RMT, H-coil	Crossover trial	FMA-LE, MAS, 10-MT, 6MWT
Rastgoo et al., 2016 [15]	7/7	54.60 ± 11.75/49.70 ± 11.00	30.2 ± 18.3/27.4 ± 20.1	*r*/sham	20 min/day, 5 days	Leg motor cortex of the unaffected hemisphere	1 Hz, 90% RMT, eight coil	Crossover trial	MMAS, FMA-LE, *H*_max_/*M*_max,_ TUG
Yijie, 2018 [20]	70/70	55.20 ± 11.50/51.30 ± 12.10	31.60 ± 11.5/51.3 ± 12.1	*r* + *R*/sham + *R*	20 min/day, 5 days	Contralateral cerebral cortex	1 Hz, 90% RMT, eight coil	Crossover trial	MAS, FMA-LE, *H*_max_/*M*_max_, TUG
Jing et al., 2018 [21]	24/24	56.55 ± 13.11/57.33 ± 12.00	3.58 ± 2.44/4.01 ± 2.89 days	*r* + *R*/*R*	15 min/day, 1 month	Primary motor cortex of the unaffected hemisphere	1 Hz, 90% RMT	Factorial trial	MAS, FMA-LE, MBI, BBS
Jing et al., 2018 [21]	24/24	56.21 ± 11.68/55.93 ± 13.88	4.33 ± 2.57/4.41 ± 2.69 days	*r* + *B* + *R*/*B* + *R*	15 min/day, 1 month	Primary motor cortex of the unaffected hemisphere	1 Hz, 90% RMT	Factorial trial	MAS, FMA-LE, MBI, BBS
Yang et al., 2015 [22]	60/60	58.7 ± 3.5/59.2 ± 3.3	4.6 ± 1.2/4.3 ± 1.4	*r* + *R*/*r*	15 min/day, 5 days/week, 8 weeks	M1 of the affected hemisphere	2 Hz, 90% RMT butterfly shaped coils	Parallel trial	FMA, FAC, CSI, 10-MT
Hong et al., 2016 [17]	20/20	62.18 ± 13.66/61.23 ± 14.24	3.98 ± 2.05/4.61 ± 2.50 days	*r* + *R*/*R*	20 min/day, 6 days/week, 4 weeks	Primary motor cortex of the unaffected hemisphere	1 Hz, 90% RMT circular coil	Factorial trial	MAS, FMA-LE, MBI
Hong et al., 2016 [17]	20/20	61.99 ± 15.02/60.89 ± 15.16	4.02 ± 3.17/4.35 ± 3.28 days	*r* + *B* + *R*/*r* + *B*	20 min/day, 6 days/week, 4 weeks	Primary motor cortex of the unaffected hemisphere	1 Hz, 90% RMT circular coil	Parallel trial	MAS, FMA-LE, MBI
Huayao et al., 2019 [23]	47/45	43.33 ± 9.18/44.33 ± 9.94	─────	*r* + *F*/sham + *F*	20 min/day, 5 days/week, 4 weeks	M1 of the affected brain hemisphere	1 Hz	Parallel trial	MAS, FMA-LE, MEP

*r*: rehabilitation; sham: sham rTMS; c: cycling; *B*: BTX-A; *R*: rehabilitation; *F*: FES (functional electrical stimulation).

**Table 2:** PE-Dro scale of the included study.

Study	Item 1	Item 2	Item 3	Item 4	Item 5	Item 6	Item 7	Item 8	Item 9	Item 10	Item 11	Total
Chieffo et al., 2020 [19]	1	1	0	1	1	1	1	1	0	1	1	9
Rastgoo et al., 2016 [15]	1	1	0	1	1	0	0	0	1	1	1	7
Yijie, 2018 [20]	1	0	0	1	1	0	0	1	1	0	1	6
Jing et al., 2018 [21]	1	1	0	1	1	0	0	1	1	1	1	7
Yang et al., 2015 [22]	1	1	0	1	0	0	0	1	1	1	1	7
Hong et al., 2016 [17]	1	1	0	1	0	0	1	1	1	1	1	8
Huayao et al., 2019 [23]	1	0	0	1	0	0	1	1	1	1	1	7

## Data Availability

All the data supporting this systematic review and meta-analysis are included in this study.
